# Risk factors of falls in elderly patients with visual impairment

**DOI:** 10.3389/fpubh.2022.984199

**Published:** 2022-08-22

**Authors:** Shuyi Ouyang, Chunwen Zheng, Zhanjie Lin, Xiaoni Zhang, Haojun Li, Ying Fang, Yijun Hu, Honghua Yu, Guanrong Wu

**Affiliations:** ^1^Department of Ophthalmology, Guangdong Eye Institute, Guangdong Provincial People's Hospital, Guangdong Academy of Medical Sciences, Guangzhou, China; ^2^Graduate School, Shantou University Medical College, Shantou, China; ^3^School of Medicine, South China University of Technology, Guangzhou, China

**Keywords:** visual impairment, elderly patients, falls, risk factor, prediction tool

## Abstract

**Objective:**

To examine the risk factors for falls in elderly patients with visual impairment (VI) and assess the predictive performance of these factors.

**Methods:**

Between January 2019 and March 2021, a total of 251 elderly patients aged 65–92 years with VI were enrolled and then prospectively followed up for 12 months to evaluate outcomes of accidental falls *via* telephone interviews. Information of demographics and lifestyle, gait and balance deficits, and ophthalmic and systemic conditions were collected during baseline visits. Forward stepwise multivariable logistic regression analysis was performed to identify independent risk factors of falls in elderly patients with VI, and a derived nomogram was constructed.

**Results:**

A total of 143 falls were reported in 251 elderly patients during follow-up, with an incidence of 56.97%. The risk factors for falls in elderly patients with VI identified by multivariable logistic regression were women [odds ratio (OR), 95% confidence interval (CI): 2.71, 1.40–5.27], smoking (3.57, 1.34–9.48), outdoor activities/3 months (1.31, 1.08–1.59), waking up frequently during the night (2.08, 1.15–3.79), disorders of balance and gait (2.60, 1.29–5.24), glaucoma (3.12, 1.15–8.44), other retinal degenerations (3.31, 1.16–9.43) and best-corrected visual acuity (BCVA) of the better eye (1.79, 1.10–2.91). A nomogram was developed based on the abovementioned multivariate analysis results. The area under receiver operating characteristic curve of the predictive model was 0.779.

**Conclusions:**

Gender, smoking, outdoor activities, waking up at night, disorders of balance and gait, glaucoma, other retinal degeneration and BCVA of the better eye were independent risk factors for falls in elderly patients with VI. The predictive model and derived nomogram achieved a satisfying prediction of fall risk in these individuals.

## Introduction

The population with visual impairment and blindness is projected to be more than doubled by 2050 as a result of shifting demographics and global aging, reaching over 1.5 billion (1). Previous research demonstrated that vision loss is often associated with worse subjective wellbeing (2), poorer daily functions (3), and adverse health consequences among the elderly (4).

Falls are one of the most common leading causes of unintentional injury and death (5). Globally, fall-related death rates are the highest among people with visual impairment and adults aged over 50 (6). The increase in fall risk in the elderly is unavoidable because of the deterioration with age in physical, visual, and cognitive functions. Visual impairment is reported to be an independent risk factor for falls and fractures among older people (7, 8). Therefore, older people with visual impairment should be the main targets of fall risk assessment.

Falls are often caused by more than one risk factor, and this is particularly the case for older people with visual impairment (7). People with poor vision can easily lose stability and change different gaits to avoid obstacles, thus increasing the risk of slips, trips, and falls (9). Even though older people with visual impairment may share risk factors that were previously identified in the general population with those without, they also have their own independent risk factors. Besides, since the main causes of visual impairment, such as cataract (10), glaucoma (11), and retinal degenerations (12), are found associated to fall risk, their respective effects on falls are potentially significant but still understudied.

Even though some patients with eye diseases have undergone ophthalmic surgeries, their visual impairment might still endure. For these patients, post-discharge fall risk assessment and prevention are of great significance in improving patients' quality of life and reducing fall-related injuries and deaths. Although many fall risk assessment scales have been widely used in clinical practice to highlight high-risk groups, to our best knowledge, there is no scale developed specifically for the elderly with visual impairment. Besides, only a handful of cross-sectional studies or studies involving adults have discussed the risk factors for falls in patients with visual impairment (13, 14). Moreover, the associations of eye diseases, ophthalmic surgeries, and other eye-related factors with incident falls in patients with visual impairment are still unclear.

Therefore, this study included elderly patients with visual impairment. In addition to demographics and lifestyle, gait and balance deficits, and systemic conditions, we also examine the associations between eye-related factors and incident falls in visual impairment patients. Based on the result of regression analysis, we further assessed the predictive performance of these factors, and derived a fall risk prediction nomogram for the elderly patients with visual impairment.

## Methods

### Design and population of the study

This longitudinal study recruited a total of 251 elderly patients with visual impairment, aged 65–92 years. The study was conducted in the Department of Ophthalmology, Guangdong Provincial People's Hospital from January 2019 to March 2021. The procedures adhered to the ethical standards of the Research Ethics Committee of Guangdong Provincial People's Hospital (registration number: KY2020-651-01) and the Helsinki Declaration. Informed consent was obtained from all participants.

Inclusion criteria for patients were diagnosed with visual impairment at discharge and aged ≥ 65 years. Visual impairment was defined as the best-corrected visual acuity (BCVA) worse than 0.3 logMAR units (Snellen 20/40) in the better-seeing eye. All patients were able to cooperate in completing the questionnaire survey and follow-up. The exclusion criteria for patients were with (1) falls caused by compression; (2) stroke-related impaired physical mobility; (3) senile dementia; (4) severe systemic diseases (e.g., cerebral infarction, myocardial infarction, heart failure, or connective tissue disorder); (5) endpoints such as loss of follow-up or death occurred during follow-up.

### Data collection

The patient information was collected through a detailed interviewer-administered questionnaire during ophthalmic admissions, including demographics and lifestyle, gait and balance deficits, and ophthalmic and systemic conditions. The questionnaires were orally administered to each patient by two trained ophthalmic nurses and the answers were recorded accordingly in data collection sheets. In each survey, the interviewers adopted the same questioning method to reduce bias. In addition, blood test results and surgeries-related data were manually extracted from the electronic medical record system. All the data were double-checked by a professional ophthalmologist (CZ).

The body mass index (BMI) was measured as weight in kilograms divided by the square of height in meters. Smoking status was classified into never-smokers and smokers (including former and current smokers), and alcohol consumption was defined based on self-reported history of alcoholic beverage ingestion. Sleep duration was determined by the average sleep duration over the past month, while nocturnal awakening was further classified as never/occasional or frequent based on self-report. Outdoor participation was referred to the times of outdoor physical activities and exercise over the past 3 months.

Diabetes was defined as a random glucose level ≥ 11.1 mmol/L, glycated hemoglobin (HbA1c) ≥ 6.5%, use of diabetic medication, or a self-reported history. Hypertension was defined as systolic blood pressure (BP) ≥ 140 mmHg, diastolic BP ≥ 90 mmHg, anti-hypertensive drug use, or a self-reported history. Cardiovascular disease (CVD) was defined based on a self-reported history of angina, stroke, or heart attack. Laboratory tests included glycated hemoglobin, platelets, and serum sodium, potassium, and calcium levels. Health status was divided into good and bad based on the responses of patients when asked “what is your health status as compared to the general health conditions of age-matched individuals”.

Difficulty walking was defined as the inability to walk properly due to abnormal and uncontrollable walking patterns. Weakness was defined as self-reported weakness or weakness of the lower extremities. States of balance and gaits were assessed and then classified into normal and abnormal based on the following criteria. Normal balance and gait required natural gaits with eyes open, standing unsupported with eyes closed for over 10 s (safely), and looking behind from both sides with satisfying weight shifts. Meanwhile, disorders of balance and gait (abnormal) represented unsteady gaits and/or standing with eyes open, standing unsupported with eyes closed for <3 s, or failure to look behind (i.e., from one side only, turning sideways only, supervision needed, and assistance needed). Sit-to-stand test was performed to classify patients into independence in standing up and inability to stand up without assistance.

### Ophthalmic variables

All patients underwent comprehensive ophthalmic examinations, including (measured on a decimal chart and presented as logMAR), autorefraction, intraocular pressure, slit-lamp examination, and color fundus photography. We also collected information of ophthalmic condition, including glare, visual clarity, use of eye drops, history of eye disease, as well as ophthalmic surgeries. Our study adopted the WHO criteria (International Classification of Diseases 11th edition criteria for vision loss) for visual impairment, i.e., BCVA worse than 0.3 logMAR (Snellen 20/40) in the better eye (15).

### Incidence of fall

After discharge, patients were prospectively followed up for 12 months to determine outcomes of accidental falls by interviewing patients or their family members by telephone. In this study, a fall was defined by WHO as “an event which results in a person coming to rest inadvertently on the ground or floor or other lower level”, and a faller as an individual who had at least one fall during a 12-month follow-up.

### Statistical analyses

Baseline characteristics were tabulated for those with falls at follow-up. Continuous variables with a normal distribution were presented as mean (standard deviation), while those with a non-normal distribution were presented as median (interquartile range). Besides, categorical variables were reported as counts (percentage). Chi-square tests were used to compare categorical variables, while *t*-tests or Wilcoxon rank-sum tests were used to measure the differences in/within continuous variables.

Univariate logistic regressions were applied to identify risk factors for falls in elderly patients with visual impairment. All variables significantly associated with fall (*P* < 0.20) were included in the forward stepwise multivariable logistic regression analysis. Variables with *P*-values < 0.05 in multivariable analysis were adopted in the final predicted model that laid solid foundations for the following construction of the nomogram for fall risk prediction in elderly patients with visual impairment. Scores for each variable were calculated based on their regression coefficient values. As a result, the total score of each individual could be converted into the predicted probability of fall.

The model performance was assessed by the area under receiver operating characteristic curve (16) that reflects the accuracy of the diagnostic system. Homser-Lemeshow (H-L) was used for calibration and to measure how closely the predicted probabilities agree with the actual outcomes, and a *P*-value > 0.05 indicated good calibration. Finally, we calculated the sensitivity and specificity of the prediction model.

All statistical analyses were conducted by using Stata version 16.0 (StataCorp, College Station, TX).

## Results

### Participant characteristics

A total of 251 patients with visual impairment (57.77% women) aged 65–95 years [median (interquartile range): 78.00 (12.00)] were included in the analysis. During a follow-up of 12 months, the incidence of falls among 251 patients with visual impairment was 56.97%. Baseline characteristics and ophthalmic conditions stratified by fall status at follow-up were presented in Tables 1, 2, respectively. Fallers were more likely to be former or current smoker, frequent night awakenings, active outdoor activity, difficulty in walking, inability to stand up without assistance, weakness, disorders of balance and gait, and bad health status at baseline. Besides, fallers at baseline had worse BCVA of the better eye and a higher prevalence of cataract, glaucoma, and other retinal degenerations.

**Table 1:** Baseline characteristics stratified by fall status at follow-up.

**Baseline characteristics**	**Total**	**Fall**	**Non-fall**	***P*-value**
N	251	143 (56.97)	108 (43.03)	
**Demographic factors**				
Age (SD), year^†^	78.00 (12.00)	78.00 (12.00)	78.00 (11.00)	0.220
Gender, *N* (%)				0.164
Men	106 (42.23)	55 (38.46)	51 (47.22)	
Women	145 (57.77)	88 (61.54)	57 (52.78)	
High (SD), m^†^	1.60 (0.10)	1.60 (0.10)	1.60 (0.13)	0.426
Weight (SD), kg^‡^	59.85 (10.17)	59.36 (10.35)	60.51 (9.94)	0.373
BMI (SD), kg/m^2†^	23.00 (4.43)	23.05 (4.92)	22.97 (3.87)	0.726
Retire, *N* (%)				0.059
No	20 (5.49)	11 (6.75)	9 (4.48)	
Yes	344 (94.51)	152 (93.25)	192 (95.52)	
Live alone, *N* (%)				0.272
No	18 (7.20)	8 (5.63)	10 (9.26)	
Yes	232 (92.80)	134 (94.37)	98 (90.74)	
**Systemic condition**				
Hypertension, *N* (%)				0.873
No	113 (45.02)	65 (45.45)	48 (44.44)	
Yes	138 (54.98)	78 (54.55)	60 (55.56)	
Diabetes, *N* (%)				0.908
No	171 (68.13)	97 (67.83)	74 (68.52)	
Yes	80 (31.87)	46 (32.17)	34 (31.48)	
Cardiovascular disease, *N* (%)				0.982
No	207 (82.47)	118 (82.52)	89 (82.41)	
Yes	44 (17.53)	25 (17.48)	19 (17.59)	
Laboratory tests, *N* (%)				0.323
Normal	222 (88.45)	124 (86.71)	98 (90.74)	
Abnormal	29 (11.55)	19 (13.29)	10 (9.26)	
Health status, *N* (%)				**0.016**
Good	201 (80.08)	107 (74.83)	94 (87.04)	
Bad	50 (19.92)	36 (25.17)	14 (12.96)	
**Lifestyle factors**				
Drinking, *N* (%)				0.326
Never	207 (82.47)	115 (80.42)	92 (85.19)	
Former or current	44 (17.53)	28 (19.58)	16 (14.81)	
Smoking, *N* (%)				**0.026**
Never	216 (86.06)	117 (81.82)	99 (91.67)	
Former or current	35 (13.94)	26 (18.18)	9 (8.33)	
Sleep duration (SD), hours^†^	6.00 (1.00)	6.00 (1.50)	6.00 (1.00)	0.358
Waking up at night, *N* (%)				**0.007**
Never or occasionally	155 (61.75)	78 (54.55)	77 (71.30)	
Frequent	96 (38.25)	65 (45.45)	31 (28.70)	
Outdoor activities (SD), times/3 months^†^	0 (0)	0 (0)	0 (0)	**0.002**
**Balance-related factors**				
Difficulty in walking, *N* (%)				**0.002**
No	195 (77.69)	101 (70.63)	94 (87.04)	
Yes	56 (22.31)	42 (29.37)	14 (12.96)	
Sit-to-stand test				**0.043**
Independent stand up	235 (93.63)	130 (90.91)	105 (97.22)	
Inability to stand up without assistance	16 (6.37)	13 (9.09)	3 (2.78)	
Dizziness, *N* (%)				0.187
No	245 (97.61)	138 (96.50)	107 (99.07)	
Yes	6 (2.39)	5 (3.50)	1 (0.93)	
Weakness, *N* (%)				**<0.001**
No	194 (77.29)	99 (69.23)	95 (87.96)	
Yes	57 (22.71)	44 (30.77)	13 (12.04)	
Disorders of balance and gait, *N* (%)				**<0.001**
No	181 (72.11)	89 (62.24)	92 (85.19)	
Yes	70 (27.89)	54 (37.76)	16 (14.81)	

Data are mean (standard deviation), or median (interquartile range), or N (%). BMI, body mass index. All P-values were calculated using the ^‡^t-test or ^†^Wilcoxon rank-sum tests for continuous variables and the Chi-square tests for categorical variables. Boldface indicates statistical significance with P < 0.05.

**Table 2:** Baseline ophthalmic conditions stratified by fall status at follow-up.

**Baseline characteristics**	**Total**	**Fall**	**Non-fall**	***P*-value**
Glare, *N* (%)				0.361
No	188 (74.90)	104 (72.73)	84 (77.78)	
Yes	63 (25.10)	39 (27.27)	24 (22.22)	
Visual clarity, *N* (%)				0.099
Both sunny and rainy days	135 (53.78)	72 (50.35)	63 (58.33)	
Sunny days	107 (42.63)	63 (44.06)	44 (40.74)	
Rainy days	9 (3.59)	8 (5.59)	1 (0.93)	
The number of eye drops used^†^	2 (2)	2 (2)	2 (2)	0.504
Cataract, *N* (%)				**0.045**
No	36 (14.34)	15 (10.49)	21 (19.44)	
Yes	215 (85.66)	128 (89.51)	87 (80.56)	
Glaucoma, *N* (%)				**0.001**
No	215 (85.66)	113 (79.02)	102 (94.44)	
Yes	36 (14.34)	30 (20.98)	6 (5.56)	
AMD, *N* (%)				0.643
No	185 (73.71)	107 (74.83)	78 (72.22)	
Yes	66 (26.29)	36 (25.17)	30 (27.78)	
Other retinal degenerations, *N* (%)				**0.010**
No	222 (88.45)	120 (83.92)	102 (94.44)	
Yes	29 (11.55)	23 (16.08)	6 (5.56)	
Refractive error, *N* (%)				0.075
No	102 (40.96)	65 (45.77)	37 (34.58)	
Yes	147 (59.04)	77 (54.23)	70 (65.42)	
Intraocular lens implantation, *N* (%)				0.661
No	71 (28.29)	42 (29.37)	29 (26.85)	
Yes	180 (71.71)	101 (70.63)	79 (73.15)	
Glaucoma surgery, *N* (%)				0.463
No	247 (98.41)	140 (97.90)	107 (99.07)	
Yes	4 (1.59)	3 (2.10)	1 (0.93)	
Vitreoretinal surgery, *N* (%)				0.415
No	184 (73.31)	102 (71.33)	82 (75.93)	
Yes	67 (26.69)	41 (28.67)	26 (24.07)	
Anterior segment surgery, *N* (%)				0.648
No	240 (95.62)	136 (95.10)	104 (96.30)	
Yes	11 (4.38)	7 (4.90)	4 (3.70)	
BCVA of the better eye (SD), logMAR^†^	1.07 (0.99)	0.83 (0.45)	1.25 (1.23)	<0.001

Data are mean (standard deviation), or median (interquartile range), or N (%). BCVA, best-corrected visual acuity. All P-values were calculated using the ^†^Wilcoxon rank-sum tests for continuous variables and the Chi-square tests for categorical variables. Boldface indicates statistical significance with P < 0.05.

### Risk factors for falls in elderly visual impairment patients

The results of univariate logistic regression for fall risks in elderly patients with visual impairment were slowed in Table 3. The incident falls were associated with women [odds ratio (OR), 95% confidence interval (CI): 1.47, 0.86–2.37], retirement (0.25, 0.05–1.17), smoking (2.44, 1.09–5.46), outdoor activities/3 months (1.20, 1.01–1.43), waking up frequently during the night (2.07, 1.22–3.52), bad health status (2.26, 1.15–4.44), difficulty in walking (2.79, 1.43–5.44), inability to stand up without assistance (3.50, 0.97–12.61), weakness (3.25, 1.65–6.41), disorders of balance and gait (3.49, 1.86–6.55), cataract (2.06, 1.01–4.22), glaucoma (4.51, 1.80–11.29), and other retinal degenerations (3.26, 1.28–8.31), refractive error (4.73, 1.58–14.16), and BCVA of the better eye (1.88, 1.25–2.81).

**Table 3:** Univariate logistic regression for fall risk in elderly patients with visual impairment.

**Variables**	**Odds ratio (95% CI)**	***P*-value**
**Gender**
Men	(Reference)	
Women	1.43 (0.86–2.37)	**0.165**
**Retire**
No	(Reference)	
Yes	0.25 (0.05–1.17)	**0.079**
**Outdoor activities, times/3 months**	1.20 (1.01–1.43)	**0.036**
**Smoking**
Never	(Reference)	
Former or current	2.44 (1.09–5.46)	**0.029**
**Waking up at night**
Never or occasionally	(Reference)	
Frequent	2.07 (1.22–3.52)	**0.007**
**Health status**
Good	(Reference)	
Bad	2.26 (1.15–4.44)	**0.018**
**Difficulty in walking**
No	(Reference)	
Yes	2.79 (1.43–5.44)	**0.003**
**Sit-to-stand test**
Independent stand up	(Reference)	
Inability to stand up without assistance	3.50 (0.97–12.61)	**0.055**
**Weakness**
No	(Reference)	
Yes	3.25 (1.65–6.41)	**0.001**
**Disorders of balance and gait**
No	(Reference)	
Yes	3.49 (1.86–6.55)	**<0.001**
**Visual clarity**
Both sunny and rainy days	(Reference)	
Sunny days	1.25 (0.75–2.09)	**0.389**
Rainy days	7.00 (0.85–57.52)	**0.070**
**Cataract**
No	(Reference)	
Yes	2.06 (1.01–4.22)	**0.048**
**Glaucoma**
No	(Reference)	
Yes	4.51 (1.80–11.29)	**0.001**
**Other retinal degenerations**
No	(Reference)	
Yes	3.26 (1.28–8.31)	**0.013**
**Refractive error**
No	(Reference)	
Yes	4.73 (1.58–14.16)	**0.006**
**BCVA of the better eye**	1.88 (1.25–2.81)	**0.002**

BCVA, best-corrected visual acuity. Boldface indicates statistical significance with P < 0.20.

**Figure 1:**
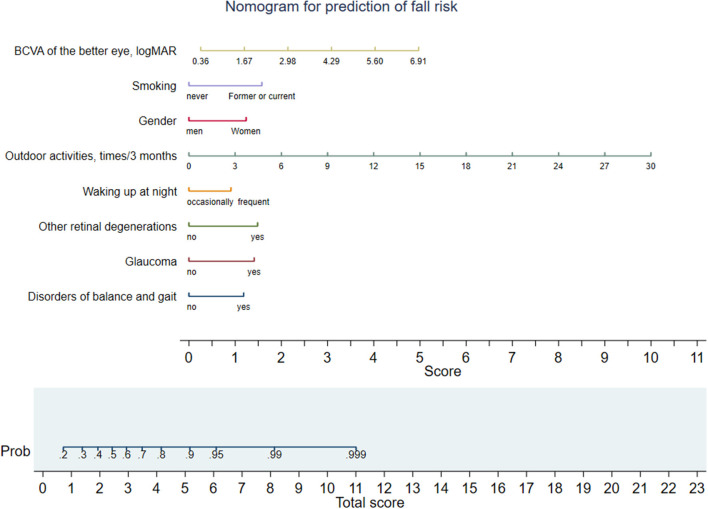
Nomogram for fall risk prediction of elderly patients with visual impairment. Different values of each variable correspond to different positions in the nomogram. Draw a line from the position of cach variable to the points axis for acquiring points of this variable. Points of different variables are summed to yield a total score that can be converted into predicted probability of fall based on the total score axis of the nomogram. BCVA, best-corrected visual acuity.

The results of stepwise multivariate logistic regression for fall risks in elderly patients with visual impairment were slowed in Table 4. The independent risk factors for falls among elderly patients with visual impairment identified by multivariate logistic regression were women (2.71, 1.40–5.27), smoking (3.57, 1.34–9.48), outdoor activities/3 months (1.31, 1.08–1.59), waking up frequently during the night (2.08, 1.15–3.79), disorders of balance and gait (2.60, 1.29–5.24), glaucoma (3.12, 1.15–8.44), other retinal degenerations (3.31, 1.16–9.43) and BCVA of the better eye (1.79, 1.10–2.91). A nomogram was developed based on these abovementioned multivariate analysis results (Figure 1).

**Table 4:** Stepwise multivariate logistic regression for fall risk in elderly patients with visual impairment.

**Variables**	**Odds ratio (95% CI)**	***P*-value**
**Gender**
Men	(Reference)	
Women	2.71 (1.40–5.27)	**0.003**
**Smoking**
Never	(Reference)	
Former or current	3.57 (1.34–9.48)	**0.011**
**Outdoor activities, times/3 months**	1.31 (1.08–1.59)	**0.006**
**Waking up at night**
Never or occasionally	(Reference)	
Frequent	2.08 (1.15–3.79)	**0.016**
**Disorders of balance and gait**
No	(Reference)	
Yes	2.60 (1.29–5.24)	**0.007**
**Glaucoma**		
No	(Reference)	
Yes	3.12 (1.15–8.44)	**0.025**
**Other retinal degenerations**
No	(Reference)	
Yes	3.31 (1.16–9.43)	**0.025**
**BCVA of the better eye**	1.79 (1.10–2.91)	**<0.001**

BCVA, best-corrected visual acuity. Boldface indicates statistical significance with P < 0.05.

### The performance of predictive model

**Figure 2:**
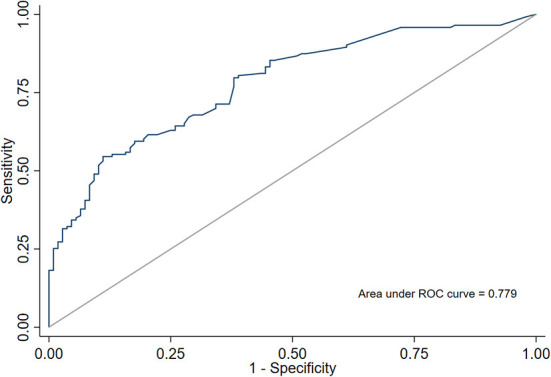
ROC curve and area under receiver operating characteristic curve (AUC) of a predictive model for fall risk in elderly patients with visual impairment.

The model performance was assessed by discrimination and calibration. The predictive model and nomogram demonstrated relatively good accuracy in estimating the risk of falls with an AUC of 0.779 (Figure 2). Hosmer-Lemeshow goodness-of-fit tests indicated good calibration (*P* > 0.05). The sensitivity and specificity of the prediction model were 0.824 and 0.650.

## Discussion

In this longitudinal study of elderly Chinese patients with visual impairment, we found that gender, smoking, outdoor activities, waking up at night, disorders of balance and gait, glaucoma, other retinal degenerations and BCVA of the better eye were independent risk factors for falls in elderly patients with visual impairment. A nomogram was developed according to the multivariate analysis results. The AUC of the predictive model was 0.779.

Female, smoking, and worse visual acuity were strongly associated with falls in in elderly patients with visual impairment. Our study also found that waking up frequently during the night increased the risk of falls in elderly people with visual impairment. Previous studies have reported that subjective poor sleep quality (17), napping (18), short sleep duration (19, 20), long sleep duration (20), and insomnia (21) were associated with an increased risk of falls and fractures (22, 23). Waking up frequently at night is one kind of sleep disorders that reduce sleep quality (24). Fracture is a risk factor for falls, and poor sleep may disrupt the diurnal rhythm of bone turnover and affect bone metabolism through other mechanisms including hypoxemia, inflammation, increased sympathetic tone, alterations in melatonin, and predispose individuals to fracture (24). Besides, sleep disorder may also lead to decreased muscle strength and physical function, as well as slower walking speed, which have been linked with falls and fractures (25, 26). Due to frequent waking up at night, the sleep quality of elderly patients with visual impairment may decrease and the body may not get adequate rest, which lead to reduced reaction ability, limb weakness, or influence bone turnover, thus increasing the risk of falling.

Our study found that disorders of balance and gait were associated with a higher risk for falls in elderly patients with visual impairment. Disorders of balance and gait are particularly important in the elderly because they compromise independence and contribute to the risk of falls and injury (27). It has been shown that when walking at a constant speed, the elderly with slower step speed and frequency, shorter stride length and stride length, longer foot support time and greater gait variability are more likely to fall (28–30). In addition, visual impairment itself may interfere with balance when walking and standing, because visual motion signals provide direct information regarding head movements (31, 32). Our study indicated that the association between disorders of balance and gait and falls were independent on visual acuity. Therefore, assessment of balance function and gait abnormalities is also an important part of determining the risk of falls in elderly patients with visual impairment.

Our study found that higher levels of outdoor activity were associated with a greater risk of falls in elderly patients with visual impairment. Activity limitation due to a fear of falling is very common in older adults with visually impairing eye disease, thus this compensatory strategy may protect against falls (33). But previous studies have demonstrated that fear of falling often results in decreased activities of daily living, leading to frailty, and decreased muscle strength and tone, which may also play a role in future falls (34–37). Besides, a U-shaped relationship was identified by a prospective cohort study between bouted physical activity and fall incidence, and both physical inactivity and high activities increase the risk of falls in older adults (38). The type of exercise was also associated with fall risk. Therefore, the effect of outdoor activities on the risk of falls in the elderly with visual impairment requires further study.

In this study, glaucoma and other retinal degenerations increased the risk for falls among elderly people with visual impairment. It has been noted that individuals with visual field damage were associated with more falls (11, 39). Inferior peripheral visual field damage and preserved inferior central visual field sensitivity were associated with increased fear of falling in glaucoma (40). At worse levels of visual field damage, patients with glaucoma demonstrated an exacerbated decline in walking speeds (ie, stride velocity and cadence) in older adults with glaucoma (41). And fear of falling and changes in gait are both risk factors for falls. Previous studies showed that age-related macular degeneration is associated with falls, postural instability, and reduced gait speed (12, 42). The differences between these studies and our results may be due to the small sample size and selection of the visually impaired population. Other retinal degenerations, such as retinitis pigmentosa, which leads to general nyctalopia and is followed by a gradual narrowing of the visual fields, may also be associated with falls in older patients. Previous studies were unable to distinguish between falls caused by visual impairment or by the eye disease itself, but our results demonstrated that glaucoma and other retinal degeneration are associated with falls. Therefore, for the elderly with visual impairment, special attention should be paid to those with glaucoma and other retinal degenerations.

In addition, to our best knowledge, there is no fall risk assessment scale that specifically targets the elderly with visual impairment to highlight the high-risk groups. Prediction models and nomograms based on predictors/risk factors of diseases can provide patients with accurate prediction tools to be used in safety management for individuals (43–45). We contributed a predictive model and nomogram based on these abovementioned risk factors for falls, and older patients with visual impairment may get a predicted probability of fall based on the total score axis of the nomogram. Future studies are needed to further validate and improve the predictive performance of this model. Our model provides a convenient method for the assessment and prevention of falls in patients with visual impairment. For patients with visual impairment who are at high risk of falling, doctors and nurses can enhance targeted education and guidance to improve patient quality of life and outcomes.

This study had uniquely examined whether a variety of fall-related factors and eye-related factors were independent risk factors for fall in patients with visual impairment, and assessed the predictive performance of these factors. The present study has several limitations. Firstly, the risk factors included in our analysis are limited. Further studies are needed to investigate the effects of other factors on the risk of falls, such as medication use. Secondly, the present study was also limited by its relatively limited sample size, and further studies with larger sample sizes would help confirm the findings of our research. Thirdly, we did not assess the performance of predictive models in patients with different degrees of visual impairment due to the small sample size. Finally, we did not compare the study on risk factors of falls in elderly peoples before and after ophthalmic surgery because of failure to review the history of falls.

In conclusion, gender, smoking, outdoor activities, waking up at night, disorders of balance and gait, glaucoma, other retinal degenerations and BCVA of the better eye were independent risk factors of falls in elderly patients with visual impairment. The predictive model and derived nomogram achieved a satisfactory prediction of fall risks in the target population. The results of this study laid solid foundations for the advanced individualization of fall interventions.

## Data availability statement

The data used during the current study are available from the corresponding authors on reasonable request.

## Ethics statement

The studies involving human participants were reviewed and approved by Research Ethics Committee of Guangdong Provincial People's Hospital. The patients/participants provided their written informed consent to participate in this study.

## Author contributions

SO, GW, and YH: conception and design. XZ, HL, and YF: data collection and collation. GW and SO: data analysis and interpretation. SO, GW, ZL, and CZ: manuscript writing. YH and HY: data interpretation and final review of the manuscript. All authors revised and approved the submitted manuscript.

## Funding

This study was supported by the Science and Technology Program of Guangdong Province (A2021003 to SO), the Nursing Research Fund of Guangdong Provincial People's Hospital (DFJH2020005 to SO), the National Natural Science Foundation of China (82171075 and 81870663), and the Science and Technology Program of Guangzhou (202206010092).

## Conflict of interest

The authors declare that the research was conducted in the absence of any commercial or financial relationships that could be construed as a potential conflict of interest.

## Publisher's note

All claims expressed in this article are solely those of the authors and do not necessarily represent those of their affiliated organizations, or those of the publisher, the editors and the reviewers. Any product that may be evaluated in this article, or claim that may be made by its manufacturer, is not guaranteed or endorsed by the publisher.
