# Corrigendum: Risk factors of falls in elderly patients with visual impairment

**DOI:** 10.3389/fpubh.2022.1087472

**Published:** 2022-12-06

**Authors:** Shuyi Ouyang, Chunwen Zheng, Zhanjie Lin, Xiaoni Zhang, Haojun Li, Ying Fang, Yijun Hu, Honghua Yu, Guanrong Wu

**Affiliations:** ^1^Department of Ophthalmology, Guangdong Eye Institute, Guangdong Provincial People's Hospital, Guangdong Academy of Medical Sciences, Guangzhou, China; ^2^Graduate School, Shantou University Medical College, Shantou, China; ^3^School of Medicine, South China University of Technology, Guangzhou, China

**Keywords:** visual impairment, elderly patients, falls, risk factor, prediction tool

In the published article, the first author's name was incorrectly written as “Ouyang Shuyi.” The correct spelling is “Shuyi Ouyang.”

The authors apologize for this error and state that this does not change the scientific conclusions of the article in any way. The original article has been updated.

